# What Is a *White Epistemology* in Psychological Science? A Critical Race-Theoretical Analysis

**DOI:** 10.3389/fpsyg.2022.861584

**Published:** 2022-05-26

**Authors:** Thomas Teo

**Affiliations:** Department of Psychology, York University, Toronto, ON, Canada

**Keywords:** critical race theory, philosophy of science, *epistemology*, knowledge, race, racism, methodologism

## Abstract

*Critical race theory* guides the analysis of the *nature* of a *white epistemology* in psychological science, the consequences for the study of race, and how scientific racism has been possible in the pursuit of knowledge. The article argues that race has not only been misused in the politics of psychology but misappropriated because of the *logic* of psychological science. The epistemic process is divided into four components to argue that naïve empiricist approaches in psychology, centered on scientific method, prevent an intricate understanding of race. Reasons for privileging method in psychology and the consequences of a *white epistemology* are discussed, including a narrow epistemic horizon and an inability to account for the temporality and contextuality of psychological phenomena. Ignorance, failure, or unwillingness to account for epistemic complexity when studying race are identified as problems. Questions about who benefits from narrow epistemologies are answered and suggestions for a broader practice of knowledge and education are provided.

The American Psychological Association (APA) released in October of 2021 an apology that acknowledges the role of APA and the discipline of psychology in “promoting, perpetuating, and failing to challenge racism” (American Psychological Association [APA], 2021a) and a resolution that aims at “dismantling systemic racism” (American Psychological Association [APA], 2021b). The apology acknowledges psychology’s history of racism, including its participation in eugenics and the maintenance of racial hierarchies, while calling for perspectives based on human rights, anti-racist approaches in all institutions, and the support for minority psychologists. While “white epistemologies” are identified as general sources of racism in psychology, without being described, the following article intends to address the question of what could be considered a *white epistemology* or in which ways the “logic of research” as the existing practice of psychological science has contributed to the problem. The issue, so goes the argument, is not just the politics and power but the *logic of psychological research*.

Acknowledging that something went wrong in psychology when dealing with race, and even more significantly, understanding the complicity and/or ignorance of (American) psychology with regard to racism in its research and practices, must be complemented by a consideration of the ways in which psychological science, as it has been taught and embodied in the practices of psychologists, has contributed to the problem. Beyond bad intentions, personal biases, or ideologically motivated psychologists, what is “happening” within psychological science itself? To answer the question of how psychological science and its logic have subsidized racism in the discipline and to argue that racism is a meaningful consequence of the logic of traditional research, I will draw on ideas from *Critical Race Theory* (CRT). Focusing on the logic of research, I intend to advance the analysis beyond problems of sampling, reporting, reviewing, and disseminating research (see Buchanan et al., 2021).

## Critical Race Theory for Psychological Science

Because of the confusion that the project of CRT evokes, it is necessary to discuss its meanings. There exist at least three different “language games” when it comes to the term *Critical Race Theory*: (a) CRT refers to an approach within legal studies that has been developed since the 1970s and 1980s; (b) CRT has been applied and extended, explicitly or implicitly, to other academic projects in the humanities, social sciences, and education; and (c) CRT is politically misinterpreted to suggest that *white* people are inherently bad or oppressive, a narrative that has gained political currency, particularly in the United States (see e.g., Hargis and Walker, 2021). This article will draw mostly on (b), which is supported by empirical evidence and able to guide further analyses.

CRT was developed by legal scholars, including Bell (1976) and Freeman (1978), as a framework for studying how race and racism play out in law and the legal system (for overviews, see Delgado and Stefancic, 1993; Crenshaw et al., 1995). An important innovation was made by the critical race theorist Crenshaw (1989), who emphasized the intersectionality of race, gender, class, and other social characteristics in legal and other social contexts (for psychology, see Rosenthal, 2016). Outside legal theory, critical ideas on race have been developed, sometimes with reference to CRT, in education (e.g., Dixson et al., 2017), sociology (e.g., Omi and Winant, 1994), historiography (Pascoe, 1996), as well as psychology (Salter and Adams, 2013; Fine and Cross, 2016; Salter et al., 2018), and other disciplines. For philosophy, Mills (1997) developed the argument that America is based on a racial contract of *white* supremacy, not only politically but also epistemologically.

Theorizing *white epistemology*, this argument draws on core tenets of CRT (for psychology see Salter and Adams, 2013; Delgado and Stefancic, 2017): (a) Although the meaning of race and racism are socially and historically constituted (“racism without races”), the effects of those constructions and racializations are tangible. The process of racialization and the social constitution of race have been shown by anthropological and historical research (e.g., Hannaford, 1996; Yudell, 2014). Thus, *Whiteness* in this article is not understood as a racial category. Similar to Mills’s (2007) argument about white ignorance, a *white epistemology* is not confined to *white* people. Indeed, one could use concomitantly the terms dominant, hegemonic, traditional, or Euro-American indigenous epistemology. The article rejects the idea that historically developed race divisions should be treated as “natural kind” categories in research, reified through results of empirically found differences (Teo, 2018).

A *white epistemology* is not inherently a problem, no more than any other indigenous epistemology, but historically has become problematic when it embodies hegemony and relates to the study of *other races*. As CRT points out (b) racism is a common practice, enacted by states, governments, and institutions (for instance, in Northern America), that reflects the economic, social, political, or cultural interests of dominant groups in society (Feagin, 2006). The argument presented here draws on knowledge that “white” people have benefitted from research on race (see Gould, 1996) and analyzes how the logic of research is tilted toward the needs and interests of dominant racialized groups in society (for *Whiteness* see also Lipsitz, 2006). The term *white epistemology* is justified when knowledge benefits one ethno-cultural group to the detriment of another.

Following the previous argument, CRT proposes that (c) race and racism are embedded in structures (e.g., American society), institutions (e.g., the legal system, education, the labor market) and life-worlds that lead to unequal outcomes for racialized people. These outcomes emerge independently of the intentions, attitudes, or behaviors of identifiable individuals. Racism is systemic (Elias and Feagin, 2016) and science is not excluded from this process if one considers science to be part of society. This article develops the idea that a *white epistemology* is embedded in the logic of psychological science. Arguably the *psychological humanities* (see Teo, 2017) have a different logic of research (Gadamer, 1960/1997) and would require a separate analysis (that is not provided here). For instance, one could identify a *white epistemology* within an ignorance (Mills, 2007) that presumes European history to be central and the standard against which all other historiographies be measured, an assumption and practice challenged by postcolonial historians (e.g., Chakrabarty, 2000).

CRT suggests that (d) the voices, perspectives, and first-person experiences of racialized people should be included in research (e.g., Collins, 1991). The following argument agrees that neglecting the voices of racialized people leads to ignorance and distortions about their lives and subjectivities. Not including the voice of the *Other* is indeed an important aspect of a *white epistemology* (see also Said, 1978/1979). However, the proposed perspective suggests that voice is necessary but insufficient should the logic of psychological research not be changed. Thus, this argument drawing on CRT investigates the degree to which a *white epistemology* is embedded in the system of psychological science and its consequences for the study of race (including scientific racism).

Arguably, the American Psychological Association [APA] (2021a,b) resolutions are drawing on ideas of CRT when understanding racism as part of an institution and when considering power in the discipline of psychology. Psychologists and researchers interested in psychology have addressed some of these critical topics from historical points of view (Tucker, 1994; Gould, 1996; Jackson and Weidman, 2004; Winston, 2004; Richards, 2012). Psychologists have also looked at the institutional structure of psychology and its manifestation in psychological research, in terms of sampling, problematic generalizations, editorial boards, and ignorance about race (Arnett, 2008; Henrich et al., 2010; Roberts et al., 2020; Buchanan et al., 2021). It is not surprising that some critical psychologists have understood the discipline and practice as a colonial project, with calls for the decolonization of psychology (e.g., Decolonial Psychology Editorial Collective, 2021).

## The Logic of (Psychological) Science

Using a rational reconstruction, the analysis begins with the question of how and in which ways epistemic injustice is embedded in methodic rationality as a standard practice of truth in psychology. The existing logic of psychological science makes it difficult and implausible, if not impossible, to understand how scientific racism (for examples see Winston, 2020a) has been possible (likewise for scientific sexism and scientific classism). To develop the argument that a *white epistemology* is inherent in the logic of psychological science, an analysis of the components of research is required. Drawing on philosophies of science, the practice of knowledge is divided into the *context of discovery, context of justification, context of interpretation*, and *context of translation*.

The distinction between the context of discovery and context of justification goes back to the logical-positivist philosopher of science, Reichenbach (1938), who studied the natural sciences when making his well-known distinction. He argued that researchers intending to reconstruct knowledge should focus on *internal* relations and not on the *external* sources of knowledge; the latter he considered the domain of sociology and psychology. The study of knowledge should focus, according to Reichenbach, on the internal structure of knowledge, on what he called the *context of justification*. The critical rationalist Popper (1935/1992) made a similar distinction between epistemology and psychology to suggest that the study of science should focus on how statements are tested or justified (deductively for Popper) while excluding questions about the psychological sources that led to “discovery.” Both Reichenbach and Popper made the argument that scientific reconstructions of science, the practices of knowledge, should focus on the context of justification, which is considered in this argument to be at the core of a white epistemology in psychological science.

Applied to psychological research, those “positivist” prescriptions meant that psychologists should attend to how they justify their knowledge claims, to whether statements have been tested, verified or falsified, and to whether they provide internally valid, reliable, objective, or generalizable statements based on empirical (preferably experimental) studies. Although the logic of research plays out differently in various academic disciplines, the core idea of this approach is that the claim to knowledge should center on methodology in a narrow sense, more aptly expressed as *method*. Scientific method is the path to knowledge and the only path to knowledge. The context of justification excludes questions about the societal, historical, cultural, interpersonal, or personal sources of knowledge and excludes the reasons why researchers are interested in what they are studying. The context of discovery was banned from epistemic debates by leaders of positivism, of naïve empiricism and in psychology. However, understanding the external sources of knowledge is particularly relevant when studying race.

With historical, sociological, and psychological studies of science pioneered by Fleck (1935/1979) and Kuhn (1962), and with the many works in *Science and Technology Studies* (STS) (e.g., Latour and Woolgar, 1979), epistemologists have learned that an assessment of knowledge requires not only an understanding of the *internal* features of a science (i.e., method) but also an understanding of the history, politics, sociology, and psychology of science (*external* features). With Kuhn (1962), one could ask whether the distinction between the two contexts (discovery and justification) is helpful, given that the actual practice of science involves continuous entanglements between the two (see also Barad, 2006). With the participation of prominent psychologists in scientific racism (see Yakushko, 2019; Winston, 2020a), the distinction between contexts makes little analytic sense (see also Tucker, 2002). The positivism dispute in Europe (Adorno et al., 1969) was grounded in the debate on how political and personal interests, social characteristics, power, money, preconceived notions, or worldviews contribute to or even constitute scientific knowledge. Critical theorists have emphasized that the narrow focus on the context of justification, considered central for a *white epistemology* in this article, limits our understanding of knowledge in substantial ways. To fully understand the substance of knowledge, including what has been studied and, more importantly, what has not been studied in the social sciences—and no less in psychology—one needs to move beyond method.

In addition to these two classical contexts, another component in the practice of research needs to be identified, one which cannot be reduced to the context of justification. Because the data (results) produced, using rigorous methods, are not the same as the interpretation of data, interpretation represents another analytic context. Interpretation is particularly important in the social sciences and in psychology where complexity is part of the subject matter’s ontology. The context of interpretation is also grounded in debates in the philosophy of science, particularly in the underdetermination thesis (Duhem, 1905/1954; Quine, 1970), according to which theoretical interpretations of results are underdetermined by data.

The discipline of psychology depends significantly on the discussion of results to make epistemic claims (see also Holzkamp, 1964/1981). Certainly, in actual research, the context of interpretation is entangled with other contexts: The interpretation of data depends not only on the data but also on programmatic commitments, historical mentalities, cultural assumptions, and societal ideas; there exists a circularity between theory and data, when theories produce particular sets of data and when data are then interpreted within those theories. For instance, a study within scientific racism produces data within this framework and data are interpreted within the same framework (see the many examples in Tucker, 1994; Gould, 1996; Winston, 2020b).

Finally, one should add a fourth component of research, which itself is the result of historical shifts in the meaning of science as an institution: The context of “translation,” or how research results and interpretations are reported, reviewed, disseminated, used, applied, or implemented in the academic and social world. In the academic world, translation with a focus on scientific impact, involves publishing one’s results in journals, books, chapters, or reports, or presenting one’s results at conferences (exclusion and inclusion come into play). Research findings must be expressed in linguistic terms using the conventions contained, for instance, within publication manuals. Increasingly there are institutional demands to inform, involve, and engage the public when translating knowledge, and scientific contributions may be assessed in terms of their *public* relevance or impact. Granting agencies and university administrators are asking researchers to target not only the academic community, but also the public, communities, or media. Indeed, measurable “impact” (academic, public, or economic) has become a significant criterion for academic success.

Psychological science is not exempt from increasing pressures to articulate the relevance of research to the public and studies in scientific racism have always had a large impact (see also Jackson and Winston, 2021). The increasing importance of the context of translation is not peripheral to the epistemological project of science, although arguably, the myth persists in psychological science that what really counts, epistemologically, is method. For people constructed through research in a negative way (as deficient, inferior, or damaged), the process of knowledge translation is crucial. A *white epistemology* means excluding or not attending to the contexts of discovery, interpretation, and translation, while at the same time ignorance is produced (see also Mills, 2007).

## Scientific Psychology’s Identity

In psychology, positivism has had an important role as many historians and theoreticians have pointed out (Tolman, 1992; Teo, 2018). Psychology was one of the disciplines that embraced positivism (Winston, 2001) and centered on the context of justification, translated as focusing primarily on methodology and method, and developing extensive, sophisticated, and complex tools for studying psychological phenomena. The focus (critics could call it obsession) on method, seemingly justifying the status of psychology as a real science, has been labeled *methodolatry* (Bakan, 1967), the *methodological imperative* (Danziger, 1985), or *methodologism* (Teo, 2005), all important dimensions of a *white epistemology*. Psychologists justify psychological knowledge as science by claiming the use of the scientific method and maintain that psychological work is scientific because of its use of method. Less discussed in the discipline is *doing justice to the object* (e.g., race) or the claim that primacy should be given to the object and not the method. One can justifiably describe this focus on method epistemologically as naïve empiricism (Teo, 2018) and one can consider it, given its history, a hegemonic or *white epistemology.*

From an epistemic point of view, a science that includes the subjects as objects of knowledge would require reflexivity regarding sources of questions, methods, interpretations, and translations, given the socio-political track records of the discipline and, for instance, the evidence from debates on intelligence and immigration in the 1920s (Gould, 1996) and, more recently, the participation of psychologists and psychological institutions in torture practices (Hoffman et al., 2015). The primacy of method in scientific psychology entails that issues that emerge in the context of discovery are not considered relevant. However, as studies on scientific racism in psychology have demonstrated, the reconstruction of political, social, historical, and economic sources of knowledge would contribute to a better understanding of psychological work (Jackson and Weidman, 2004). Arguably, even the replication crisis (Open Science Collaboration, 2015) cannot be solved with methodology alone but requires an understanding of the nature of the psychological that may be characterized by contextuality and temporality. Let me illustrate through the use of a “thought experiment”: If the Nazis had won the war and had come to dominate the rest of the world, and found empirical differences in characteristics between Aryans and segregated Non-Aryans using scientific methods, adding that many of the characteristics have high heritability estimates, then would it be fair to conclude that those differences are natural or a given? A method focused on difference (and on statistically assessing difference) would not be able to address that question or to challenge the theoretical and practical assumptions that formed the basis of the scientific study. A focus on method alone, that is, a white epistemology, would lead to misleading knowledge.

The argument that an assessment of psychological knowledge should include not only the context of justification, but also the contexts of discovery, interpretation, and translation, is not an attempt to narrow but to broaden the meaning of science by reconstructing knowledge in its full complexity. The lack of attention to all contexts limits psychological knowledge in significant ways. For instance, the 5th edition of the American Psychological Association [APA], 2001 Publication Manual argues that psychologists are “free to examine, interpret, and qualify the results, as well as to draw inferences from them” (p. 26). From the perspective of this argument, one should add that although psychologists are free, they also have an epistemic responsibility to provide good and careful interpretations, interpretations that do justice to the topic under investigation. The ability to provide good interpretations of data and results is a skill that needs to be taught, learned, and developed as much as any other epistemic skill in psychology. However, most psychology programs do not offer textbooks or courses on teaching hermeneutic skills in the contexts of interpretation, discovery, and translation. It is assumed that the context of justification (method) solves all other epistemic problems, which cements a training focus on research methods and statistics. Yet, a modern scientific psychology, beyond a *white epistemology*, needs to account for all contexts if knowledge is to remain a priority.

The focus on method seems to justify the scientific status of psychology or enables the discipline to move up in the hierarchy of sciences. However, this focus reproduces epistemic ignorance about what it means to be a science. The outcome in psychology is not a true science but a *hyperscience* that resembles a science (by imitating and developing methods) without a full account of the psychological object (Teo, 2020). Scientific method alone, the use of small or big machines, or the rhetoric of science, can only provide a semblance of science. The obsession with being acknowledged as a science has historical and cultural roots; it became evident when it was beneficial for financial and public reasons to associate academic psychology with the natural sciences and not with history, philosophy, or the humanities (Ward, 2002). Any gaze into the history of psychology supports that argument. For instance, Woodworth (1921), a participant in scientific racism, uses the terms *scientific* and [Frame4] *science* 13 times on the first page of his textbook. The pioneer of American psychology, James (1890), whose own book would be dismissed from a presentist perspective as unscientific, also claimed psychology as a real science (indeed, the *history of psychology* could be a source for discussing each context).

All pioneers of psychology struggled with identifying the subject matter of psychology, that is, with attending to ontic questions in psychology. Yet, from a positivist point of view, theorizing the subject matter of psychology should take backstage to research methods. This focus on method has led to some of the problems discussed in indigenous, cultural, feminist or critical psychologies as well as in theoretical psychology (see Teo, 2018) and also engenders the re-occurring crises debates in the discipline (see also Wieser, 2016). Attempts to transform traditional criteria such as validity into *psychopolitical validity* (Prilleltensky, 2008), or accounting for the degree to which an intervention captures both the psychological and the political, are rare. The apparent increasing usage of qualitative methods in psychology expands the range of phenomena that count as scientific (Gergen et al., 2015). However, qualitative methods, from the perspective of this argument, are not superior and require the same ontic and epistemic discussions as quantitative methods and may have their own *white epistemic* assumptions (see also Chauhan and Sehgal, 2022). To be fair, qualitative researchers emphasize the need to add more epistemic dimensions to the assessment of knowledge, including reflexivity (e.g., Finlay and Gough, 2003).

When it comes to issues of race, and beyond a *white epistemology*, a complex understanding of the scientific process must include not only a concentration on method but must pose questions about the financing of studies, about their intent and purpose, and about the motivation for the interest in racialized group differences. More generally, such an understanding must address when, where, and how the concept of “race” (or intelligence, etc.) and its study emerged; the economic and cultural interests that engendered such studies; and social and political interests (context of discovery). This does not mean ignoring the methodological and methodic shortcomings of such studies (context of justification). An understanding of the scientific process must also include an analysis of the quality of interpretations and the relationship between theory and data and must emphasize that results do not determine interpretations (context of interpretation). Such an analysis may focus particular attention on forms of *epistemological violence* as practices that are executed when academic interpretations of empirical results implicitly or explicitly construct the “Other” as problematic or inferior, even though alternative interpretations exist (Teo, 2008). Finally, a thorough understanding of research on races should include an analysis of explicit or implicit recommendations, concrete applications, and discourses about racialized life for the public (context of translation) (e.g., should different races really be sent to different schools?).

## Problematics for the Study of Race in Psychological Science

Engaging CRT means to understand how scientific racism and racism have been produced, and cannot be avoided in the traditional logic of research on race with its focus on method. Drawing on the discussions above, three problematics emerge for understanding *white epistemology*. The focus on method leads to (a) a *narrow horizon* that excludes discussions and reflections about all components of research that must come into play when race is studied. For a scientific understanding of race or race differences, knowledge from a variety of disciplines, including history, sociology, anthropology, political science, legal studies and the philosophy of science, are required (see also Nelson et al., 2013; Bonam et al., 2019). Excluding these fields may produce limited knowledge and ignorance in scientific psychology. Thus, whenever race is used as a variable in psychological research, a move from the narrow horizon of method to all contexts that are relevant in knowledge-making is necessary.

It is fair to ask whether this narrow horizon means that a *white epistemology* permeates scientific psychology generally, or only when race is included in its studies, or only when working from the perspective of scientific racism. Generally, one can argue that a hegemonic (from a descriptive point of view) or *white* (from a historical point of view) epistemology infuses the whole discipline. Because Western culture frames the discipline, it is reasonable to ask to what degree psychology has ethnic, cultural or colonial biases, not only when it comes to studies that include race, but in basic psychological research that attempts generalizations (Teo and Febbraro, 2003). Whiteness, not understood here as a racial but as a sociological category, has permeated knowledge in psychology, including cognitive psychology and perception research (for examples see Henrich et al., 2010). Thus, it may not be racism in a narrow sense but rather a sense of Western centrality and/or superiority that guides assumptions about generalizability (as a reaction we find attempts to develop and justify an African psychology; see Nwoye, 2015). It is not always a racial, but more recently, a cultural project that assumes the superiority of Western modes of psychological thinking (Teo, 2022). From this perspective it is evident that a *white epistemology* needs to be problematized in the study of race and it becomes toxic in scientific racism.

An analysis of scientific racism should focus on the conditions that have made this project possible. Because the argument applies to psychological science as a system, historical examples would deflect attention away from the academic discipline to individual actors, which would undermine a core tenet developed in CRT. However, to elucidate the point, two examples that corroborate the logic of scientific racism are mentioned. The eugenicist Charles Davenport (1866-1944) illustrates perfectly how the focus on method misses the complexity of the problem. Davenport and Steggerda’s (1929) book on “race mixture” appears to be a prime example of pure objective empirical science, with hundreds of tables, figures, and numbers, and using batteries of tests, including psychological ones, coming to the scientific conclusion that “intermingling” would be bad.

In psychology, J. Philippe Rushton’s (1943-2012) work can be considered paradigmatic for scientific racism. His empirical studies on “three major races” and claims that certain races are more aggressive, less intelligent, and less law-abiding than others (Rushton, 1995) – using traditional psychological methods and instruments, without discussing the contexts of discovery (e.g., worldview), interpretation, and translation, and not including the voices of the *Other* who are rendered damaged and deficient through his ideas – represent an ideal form of *white epistemology*. It is for this very reason, a lack of understanding of the complexity of the problem of race, that Rushton’s papers in *Psychological Reports* have been retracted (Retraction Notice, 2021).

Hiding behind a horizon based on methodologism shows limitations when complexity is reduced to the point of distortion. If one finds, within the logic of scientific psychology, differences between two racialized groups, there is nothing in method itself that prevents one from theorizing, interpreting and concluding that those differences are natural. This logic explains how naïve empiricism was able to produce racist research (and biased research on other social characteristics) that was peer-reviewed and published but is unable to address the socio-historical constitution of those social characteristics. The latter would entail posing questions about interests in scientific racist research or the degree to which scientific racism is a *Weltanschauung* oblivious to counterevidence (Winston, 2020b). Ideological or incompetent interpretations that suggest that within-group heritability estimates can be used to explain between-group differences still need to be addressed (see Tucker, 1994).

Overall, the history of psychology demonstrates that naïve empiricist psychology has not only been unable to prevent racist (or sexist and classist) research but has encouraged it within its logic. Challenges to scientific racism have stemmed from expanding the narrow horizon of method. The call for decolonizing psychology follows from such experiences (Decolonial Psychology Editorial Collective, 2021). Decolonial and anti-racist work (e.g., Jones, 2018) is possible when race is not treated as a natural kind entity, but rather as an entity that has social, cultural, and historical dimensions.

The second issue that has plagued empiricist psychology as a *white epistemology* is (b) the *status quo supporting* role of scientific psychology. If psychology does not specifically include an anti-racist or decolonial position and instead focuses primarily on method, then it is inevitable that research in psychology reproduces the *status quo*. If one lives in a racist society, then empirical (scientific) results will reflect that racist society. *Status quo* research could find that group X has lower scores on intelligence tests than group Y, or lower scores on motivation, achievement-orientation, and so on, without tracing the entanglement of the history of race, racism, intelligence testing, and politics. Such research on *status quo* differences then reinforces racist thinking or the problematization of the group that has been construed as inferior from the beginning (this can even happen with good intentions as textbooks show). In Northern America, *status quo* supporting research reinforces Whiteness, thus assisting the interests and needs of groups that are already dominant in society.

More generally, one could argue that psychology has problems with a broad understanding of temporality. Psychology has methods that account for temporality in longitudinal research or in pre- and post-test studies. Such methods can account for developmental changes in perception, cognition, identity, affects, and so on, over a lifespan. But beyond evolutionary, age-based or situational temporality, psychology needs to account for historical changes and the ways in which history constitutes and shapes mental life (Pettit and Hegarty, 2014). History is full of psychosocial content and to understand that content for subjectivity, psychology must encompass not only the psychological sciences but also the psychological humanities (Teo, 2017). Race and racism cannot be fully understood without an understanding of the history of race and racism in various locations (see also Nelson et al., 2013). The exclusion of such histories produces ignorance about the entanglement of historical, social, and cultural contexts for a seemingly simple variable. The focus on empirically found differences, without accounting for political, economic, or cultural developments, neglects important dimensions of mental life. In short, scientific methods, for all their strengths, do not account for histories of oppression, marginalization, extermination, violence, injustice, and unequal power. Historically constituted realities such as slavery and institutional discrimination have produced different life-worlds for ethnic groups in Northern America.

Historical thinking allows for a vision not only of what is, but also of what is possible. Beyond descriptive questions of what is possible are normative questions of what should be. It is not uncommon in many scientific disciplines, from medicine to climate science, to combine descriptions, predictions, and normative reflections. Yet, good normative reflections require a deep understanding of knowledge in the psychological humanities (e.g., philosophy). Arguably, a broader horizon on psychosocial issues (including race) sets the conditions for the possibility of a fuller discussion of normative issues than a narrow horizon. To be fair, psychology has put forth concepts and methods for future possible developments. Such concepts and methods range from Vygotsky’s (1978) *zone of proximal development*, which attempts to assess what is possible for an individual, to *Participatory Action Research* (Fine and Torre, 2021), which was developed in communities to study not only what has been, but also what can be changed and achieved when people act together, and which considers marginalized persons as co-researchers. Yet, there is no place in the context of justification to consider what is possible or what justice could mean when it concerns issues of race.

Temporality needs to be accounted for, as does (c) *contextuality.* Psychological concepts, theories, methods, measures, and practices have a cultural dimension and they reflect that culture, which could be labeled an indigenous challenge to the discipline (see also Sundararajan et al., 2020). To be sure, scientific psychology has developed cross-cultural tools to investigate differences or similarities, but culture also includes the cultural constitution of scientific concepts, theories and methods. It would be strange to argue that there are cultural differences, but that they do not apply to psychological science itself. Indeed, the idea that knowledge should focus on the context of justification and that psychology should focus on method is itself a cultural (and historical) product (thus, a *white epistemology*). Although scientific methods may find empirical differences between group X and group Y, the reasons for the differences or the meaning of these differences cannot be answered within the traditional scientific method. Interpreting differences without accounting for temporality or contextuality can lead to a banality of violence.

It is evident that scientific psychology has a hegemonic European and American history and culture (Walsh et al., 2014) and that some Western countries have been reconstructed as dominant sources of academic psychology, although there have been differences between German, British or French models of doing psychology (Danziger, 1990). Arguably, scientific psychology reflects those cultures and traditions, and psychology has an indigenous *white* dimension in those very contexts, which has been distributed to the rest of the world (where they have been accepted, modified, or rejected). Thus, it is legitimate to ask to what degree Western psychology sees differences, competencies, or performances not only from the perspective of the West, but from a perspective that reflects the intellectual, economic or social interests of groups or individuals in those cultures. More radically, following CRT, one can ask whether scientific psychology is perpetuating white supremacy or the degree to which psychology represents the need for the cultural supremacy of the West—particularly the cultural supremacy of the United States, which has had such a large impact on the field of psychology (see also Liu et al., 2019).

An analysis of cultural, economic or social interests, sometimes shared by individual researchers, and issues concerning cultural supremacy, needs to include knowledge of cultures, ethnicities, races or other groups, and cannot be conducted within the context of justification. To make it clearer: Method *prevents* the asking of such questions if they are not posed as empirically testable hypotheses. Yet, such an analysis is required, given the track record of psychology with scientific racism. Equally so, issues of cultural supremacy cannot be solved within the scientific method but must rely on knowledge from the humanities and other social sciences, including critical race theories. Processes of *Othering* or of *Inferiorizing the Other*, regardless of intent, requires historical, political, and cultural knowledge that cannot be found within scientific psychology alone (although research on racism in scientific psychology can assist this project).

Some have made the argument that the process of *inferiorizing* in psychology is no different from that in other disciplines. However, other disciplines do not make the same kinds of inferiorizing interpretations as are made within psychology. For example, if technical advances in physics are made by a group from a particular geographical region, this does not mean that groups from other geographical regions are inferior, that is, it is not suggested within the discipline of physics that other groups are inferior because of a lack of similar technological advances. That type of interpretation is already the work of scientific racism or culture-supremacy. In psychology, an analysis of the mental life of another culture from the perspective of a dominant culture has an inherent cultural problem, even without invoking empirical differences, ranking and quantification. An understanding of mental life, as some of the pioneers of the discipline understood (Dilthey, 1957), requires an understanding of customs, traditions, and the social system. It also requires reflexivity because of the looping effects and the *human kind* qualities of psychological concepts (see Hacking, 1994) and the entanglement of the object with the subject of research. It requires reflexivity on the culture and subculture of the researcher, which may draw on the psychology, sociology, and history of science (see O’Doherty et al., 2019). For an assessment of the quality of psychological research, the inclusion of all contexts of research is required, arguably more so than in other disciplines.

## Conclusion

The logic of traditional psychological research, often combined (but not always) with a *Weltanschauung* (Winston, 2020b), supports the *status quo* and is unable to address the power of racism in the discipline and profession. This logic is grounded, explicitly or implicitly, in the focus on the context of justification that conceals the importance of other contexts. In elevating method, traditional psychological science is unqualified to address scientific racism. In not moving outside method, psychological science may participate in systemic racism in the conduct of research on races. In contrast to the traditional approach of psychological science (as a *white epistemology*), the study of race in psychology needs to incorporate a discussion of the contexts of discovery, interpretation, and translation. This involves the integration of knowledge from history, culture, politics, sociology, philosophy, economics, anthropology and other disciplines (e.g., genetics) about race. To be clear: The argument advanced here does not suggest that assessing issues within the context of justification, such as methodic quality in research, is irrelevant. Rather, the issue is that method is insufficient when it comes to issues of race. I submit that most researchers recognize intuitively the need for epistemic complexity, and that topics such as race require the psychological sciences as well as the knowledge produced by the psychological humanities, including the critical, postcolonial, and anti-racist ones, but this is hardly expressed in textbooks on research practices.

Doing justice to the topic of race in research requires not only changes in research practices but also changes in education and training. The teaching of psychology must move beyond an exclusive focus on method and must encourage critical thinking not only as a methodological virtue but in relation to all contexts of research, as well as in regard to reflexivity about one’s own assumptions in science and about race. This requires teaching a model of psychological science in which method does not have primacy but which also brings social, political and financial interests, and the power of/in interpretation and translation to the foreground. Education on the history of science, the history of psychology, the philosophy and sociology of science, and hermeneutics, among other humanities, would provide a general and broad knowledge base. Yet, these are exactly the courses that have been considered superfluous in scientific psychology.

Scholars have recommended the decolonization of psychology and an active anti-racist stance to overcome the racism of psychology and to avoid invoking superiority or inferiority in discussions of race. Critical race theory in a narrow or broad sense remains an important resource for thinking about race and its implications when studying psychological phenomena. Reflections in this area as well as the willingness to learn about uncomfortable histories and actualities in the discipline and profession would provide the conditions for the possibility of a more responsible psychological science. In my experience of teaching the historical and theoretical foundations of psychology, the large majority of students, even when not following debates about scientific racism, are aware that method alone misses important dimensions of mental life. There is also an openness among students to learn about methods that attempt to do more justice to complex issues, that do not work with variables, but that rather seek to capture a problem in its complexity; one such example is a *circuits of dispossession* methodology that accounts for contextuality and temporality (e.g., Fine and Ruglis, 2009).

Given the complexity and the accumulated knowledge about race and racism, it may also be important to encourage epistemic modesty in education when making knowledge claims about race. This includes an analysis of the neoliberalization of academia, which revokes the idea of modesty when celebrating the marketing of research. Outrageous claims about race and violent interpretations are accompanied by academic citations and public debates (Jackson and Winston, 2021). The reward structure of academia adds to the probability of epistemic grandiosity rather than modesty (Teo, 2019). From an epistemic point of view, science means doing justice to the problem of race and requires a broad horizon of many streams of knowledge, and because the development of this broad horizon is a long-term project, modesty seems appropriate from an epistemic point of view.

It is fair to ask how this argument accounts for alternative and disrupting projects (e.g., challenging racism) that have existed in the history of psychology. My answer is that those projects were possible not because but despite the existing logic of research. While scientific racism has always claimed science to bear witness, some of the most important interventions in psychology that challenged scientific racism have been based on historical and theoretical reconstructions (Chorover, 1979; Howitt and Owusu-Bempah, 1994; Gould, 1996; Richards, 2012) that used cultural, social and philosophical knowledge and material. This is not deny that methodical critiques remain significant when analyzing scientific racism. The move from studying race to studying racism (see also Samelson, 1978), for instance, the study of color-blind racism (Neville et al., 2016), has been made possible because of an expansion of the epistemic project from a narrow context to the broadening of sources, interpretations, and applications of psychology.

The argument is not a call for censorship. It is a call for a better interrogation of knowledge and race in psychological science, for a broadening of our horizons, and for a more comprehensive science of psychology. The call for epistemic responsibility when it comes to race and racism is not a demand for limiting research but rather an invitation to extend the boundaries of research. Encouraging accountability regarding the knowledge that exists on race is not about suppression, nor is CRT suspect when it serves to address such issues. For psychology, theories, concepts, and methods are needed that show the entanglement of the societal, interpersonal, and personal, and that allow us to understand the complexity of the subjective, including racialized subjectivities, the subjectivities of supremacy, and the subjectivities of researchers who conduct studies on race. Theorizing in psychology remains an important task, in relating empirical research to theories, developing new and drawing on existing theories, and connecting the general with the particular. CRT is one condition for the possibility of a psychology that moves the discipline from making racialized groups of people into problems to aiding in solving problems that racialized people encounter when living their everyday lives.

## Author Contributions

The author confirms being the sole contributor of this work and has approved it for publication.

## Conflict of Interest

The author declares that the research was conducted in the absence of any commercial or financial relationships that could be construed as a potential conflict of interest.

## Publisher’s Note

All claims expressed in this article are solely those of the authors and do not necessarily represent those of their affiliated organizations, or those of the publisher, the editors and the reviewers. Any product that may be evaluated in this article, or claim that may be made by its manufacturer, is not guaranteed or endorsed by the publisher.
